# Persistent Stapedial Artery Encountered during Cochlear Implantation

**DOI:** 10.1155/2022/8179062

**Published:** 2022-02-22

**Authors:** Holly Jones, Justin Hintze, Adrien Gendre, Clifton Wijaya, Fergal Glynn, Laura Viani, Peter Walshe

**Affiliations:** National Hearing Implant and Research Centre (NHIRC), Beaumont Hospital, Dublin 9, Ireland

## Abstract

**Objectives:**

Persistent stapedial artery (PSA) is a rare congenital anomaly that can complicate middle ear surgery.

**Methods:**

We present the case of a 25-year-old male who underwent right-sided cochlear implantation. A PSA was encountered lying over the middle promontory intraoperatively.

**Results:**

The PSA was carefully lifted off the middle ear promontory using a Hughes elevator to divide adhesions and delineate the artery. The implant electrode was placed through the round window niche in the usual fashion. Tragal cartilage and fibrin glue were used to control the trajectory of the electrode.

**Conclusion:**

Cochlear implantation can be performed safely in patients with PSA.

## 1. Objectives 

Persistent stapedial artery (PSA) is a rare congenital anomaly that is usually asymptomatic but may be encountered and complicates middle ear surgery. The stapedial artery arises from the 2^nd^ branchial arch at 4-5 weeks of gestation and supplies most of the nonneural structures of the head and neck. By ten weeks of gestation, the stapedial artery atrophies and the external carotid take over the function as the primary arterial supply to the head and neck.

Failure of the stapedial artery to degenerate at this time results in a PSA with an estimated prevalence of 0.02–0.48% [1, 2].

In the past, encountering a PSA during middle ear surgery would result in the discontinuation of the operation. This was due to concern regarding difficulty to control hemorrhage and distal ischemia to cochlear structures. Specific to cochlear implantation, the possibility of the electrode eroding the PSA remains a concern.

We present a case of successful cochlear implantation in a patient with a PSA.

## 2. Methods

We present the case of a 25-year-old male who underwent right-sided cochlear implantation. He had a history of bilateral profound hearing loss. He underwent a preoperative MRI internal acoustic meatus (IAM) and was deemed a good candidate for implantation.

The patient was placed under general anesthesia, and facial nerve monitoring was used.

**Figure 1:**
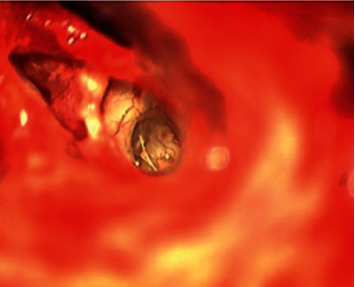
Persistent stapedial artery (x) lying over the middle ear promontory.

An aberrant persistent stapedial artery (PSA) was encountered lying over the middle ear promontory (Figure 1). The PSA was lifted carefully off the promontory. The electrode was inserted into the round window in the usual fashion. Tragal cartilage was harvested with perichondrium on both surfaces. The graft was placed between the electrode below and the PSA above. The cartilage graft was small but sufficient to ensure that the electrode array did not come into direct contact with the artery. Subsequently, the middle ear was filled with Tisseel, a fibrin sealant, to hold the graft in place and control the trajectory of the electrode to ensure that nothing shifted during the healing process.

Successful insertion of the cochlear implant was confirmed, and NRT was successfully performed.

## 3. Results

The patient recovered well and was discharged home on the first postoperative day. He was reviewed in the clinic two weeks postoperatively, and his cochlear implant was activated.

## 4. Discussion

Persistent stapedial artery (PSA) is a rare anomaly with an estimated prevalence of 0.02–0.48% [2]. It is most often a benign finding, but when encountered during middle ear surgery, it presents a surgical challenge. The stapedial artery is an embryologic structure derived from the second branchial arch that supplies the head and neck until 10 weeks of gestation. Usually, at this point, the cartilage of the stapes prevents further growth of the artery and its distal anastomosis with the external carotid leads to its eventual involution. Failure of this usual regression leads to a PSA [1].

When a PSA occurs, it usually gives rise to the middle meningeal artery and the foramen spinosum is absent ipsilaterally [3]. The artery enters the facial canal via the obturator foramen of the stapes. In normal stapedial artery involution, the obturator foramen remains as the only evidence of its existence [4]. The PSA then travels anteriorly through the facial canal to exit just before the geniculate ganglion and enters the middle cranial fossa as the middle meningeal artery [3].

The diagnosis of a PSA is usually made at the time of surgery. The anomaly may also be discovered on CT temporal bone; however, given that the vessel is so small, it may not be identified. On imaging, the tympanic segment of the facial canal may be enlarged as the PSA passes along the facial nerve. This finding could also represent a facial nerve tumor. There may also be a separate canal parallel to the facial nerve. An aberrant origin of the middle meningeal artery and an absent foramen spinosum may also alert a radiologist to a PSA [5].

In the past, when a PSA was incidentally discovered during middle ear surgery, the surgery was commonly aborted. This was due to the potential for uncontrolled bleeding and distal ischemia to structures due to injury or ligation of the artery [6]. Normally, the facial nerve receives its arterial supply from the petrosal artery, a branch of the middle meningeal artery, but this may instead arise from the PSA when present [7]. Theoretically, the patient could develop facial paralysis, hemiplegia, auditory impairment, or vestibular impairment if the PSA was disrupted. However, despite this, the best evidence suggests middle ear surgery can be performed safely in patients with PSA without neurological sequelae.

To our knowledge, this is only the second report published of a successful cochlear implant in a patient with a PSA [8]. In this case, successful implantation was achieved by the usual postauricular approach, cortical mastoidectomy, and posterior tympanotomy. At this stage, the incudostapedial joint was identified. Incidentally, a PSA was identified lying over the middle ear promontory. We lifted this with a Hughes elevator and placed the electrode through the round window niche in the usual fashion. Tragal cartilage was then placed over the electrode to prevent erosion of PSA. The trajectory of the electrode was then stabilized using fibrin glue.

## 5. Conclusion

PSA precluded middle ear surgery in the past. This case demonstrates that cochlear implantation can be performed safely and successfully in patients with this anomaly.
